# Supplementary material for: Improving the continuum of care monitoring in Brazilian HIV healthcare services: An implementation science approach
Source: PLoS One. 2021 May 10;16(5):e0250060. doi: 10.1371/journal.pone.0250060 (PMC8109816; doi:10.1371/journal.pone.0250060)
Supplement: Attachment — Submitted filename: rebuttal letter.docx [file pone.0250060.s001.docx]

Dear Editor of PLOS ONE,

Thank you for the comments and suggestions made to the manuscript “Improving the continuum of care monitoring in Brazilian HIV healthcare services: An implementation science approach”, submitted to PLOS ONE. The suggestions and comments contributed to improve our paper with a view to publication in this Journal. Below we present our responses point-by-point to the comments from editors and reviewers, as well as the changes made, identified in the revised manuscript.

Best regards

Ana Paula Loch

**Editors’ comments:**

1. Please ensure that your manuscript meets PLOS ONE's style requirements, including those for file naming. The PLOS ONE style templates can be found at

<https://journals.plos.org/plosone/s/file?id=wjVg/PLOSOne_formatting_sample_main_body.pdf> and

<https://journals.plos.org/plosone/s/file?id=ba62/PLOSOne_formatting_sample_title_authors_affiliations.pdf>

**Author’s answer:** The manuscript was carefully reviewed according PLOS ONE's style requirements.

2. We suggest you thoroughly copyedit your manuscript for language usage, spelling, and grammar. If you do not know anyone who can help you do this, you may wish to consider employing a professional scientific editing service.  
Whilst you may use any professional scientific editing service of your choice, PLOS has partnered with both American Journal Experts (AJE) and Editage to provide discounted services to PLOS authors. Both organizations have experience helping authors meet PLOS guidelines and can provide language editing, translation, manuscript formatting, and figure formatting to ensure your manuscript meets our submission guidelines. To take advantage of our partnership with AJE, visit the AJE website (http://learn.aje.com/plos/) for a 15% discount off AJE services. To take advantage of our partnership with Editage, visit the Editage website ([www.editage.com](http://www.editage.com/)) and enter referral code PLOSEDIT for a 15% discount off Editage services.  If the PLOS editorial team finds any language issues in text that either AJE or Editage has edited, the service provider will re-edit the text for free.

Upon resubmission, please provide the following:

- The name of the colleague or the details of the professional service that edited your manuscript
- A copy of your manuscript showing your changes by either highlighting them or using track changes (uploaded as a *supporting information* file)
- A clean copy of the edited manuscript (uploaded as the new *manuscript* file)

**Author’s answer:** The manuscript was reviewed by EDITAGE and submitted using tracking changes as supporting information file.

3. Thank you for including your ethics statement:

"All procedures performed in studies involving human participants were in accordance with the ethical standards of the institutional and/or national research  committee  (Ethical  Committee of  the  University  of  São  Paulo -protocol  number: 3.270.762) and  with  the  1964  Helsinki  declaration  and  its later  amendments  or  comparable  ethical  standards. The study protocol was published in Brazilian  registry  of  clinical  research (UTN  Number:  U1111-1224-4363 - <http://www.ensaiosclinicos.gov.br/rg/RBR-9xrv64/)>."

a. Please amend your current ethics statement to confirm that your named institutional review board or ethics committee specifically approved this study.

b. Once you have amended this/these statement(s) in the Methods section of the manuscript, please add the same text to the “Ethics Statement” field of the submission form (via “Edit Submission”).

For additional information about PLOS ONE ethical requirements for human subjects research, please refer to <http://journals.plos.org/plosone/s/submission-guidelines#loc-human-subjects-research>.

**Author’s answer:** The IRB name was confirmed and replaced in the sections recommended.

4.Thank you for providing the following Funding Statement:

'The study was supported by the ViiV Healthcare under Grant 210027 (<https://viivhealthcare.com/en-gb/>). DS, RZ and IP received salary from ViiV Health Care and GlaxoSmithKline. '

We note that one or more of the authors is affiliated with the funding organizations, indicating the funder may have had some role in the design, data collection, analysis or preparation of your manuscript for publication; in other words, the funder played an indirect role through the participation of the co-authors.

a. If the funding organization did not play a role in the study design, data collection and analysis, decision to publish, or preparation of the manuscript and only provided financial support in the form of authors' salaries and/or research materials, please review your statements relating to the author contributions, and ensure you have specifically and accurately indicated the role(s) that these authors had in your study in the Author Contributions section of the online submission form. Please make any necessary amendments directly within this section of the online submission form.

Please also update your Funding Statement to include the following statement: “The funder provided support in the form of salaries for authors [insert relevant initials], but did not have any additional role in the study design, data collection and analysis, decision to publish, or preparation of the manuscript. The specific roles of these authors are articulated in the ‘author contributions’ section.”

If the funding organization did have an additional role, please state and explain that role within your Funding Statement.

**Author’s answer:** The authors from funding organization played a role in the study design, decision to publish and or preparation of the manuscript. The author’s role was updated in the online submission form.

b. Please also provide an updated Competing Interests Statement declaring this commercial affiliation along with any other relevant declarations relating to employment, consultancy, patents, products in development, or marketed products, etc.

Within your Competing Interests Statement, please confirm that this commercial affiliation does not alter your adherence to all PLOS ONE policies on sharing data and materials by including the following statement: "This does not alter our adherence to PLOS ONE policies on sharing data and materials.” (as detailed online in our guide for authors http://journals.plos.org/plosone/s/competing-interests). If this adherence statement is not accurate and there are restrictions on sharing of data and/or materials, please state these. Please note that we cannot proceed with consideration of your article until this information has been declared.

Please know it is PLOS ONE policy for corresponding authors to declare, on behalf of all authors, all potential competing interests for the purposes of transparency. PLOS defines a competing interest as anything that interferes with, or could reasonably be perceived as interfering with, the full and objective presentation, peer review, editorial decision-making, or publication of research or non-research articles submitted to one of the journals. Competing interests can be financial or non-financial, professional, or personal. Competing interests can arise in relationship to an organization or another person. Please follow this link to our website for more details on competing interests: http://journals.plos.org/plosone/s/competing-interests

**Author’s answer:** As recommended, the competing Interests Statement was added on the manuscript. Changes are available bellow:

***Competing Interests***

*The commercial affiliation from authors DS, RZ and IP does not alter our adherence to  PLOS ONE policies on sharing data and materials.*

**Reviewers' comments:**

**Reviewer #1**: In general, the manuscript is technically sound. It reports on an implementation study to improve use of the Brazilian clinical monitoring system (SIMC) in service settings to identify gaps in the HIV care continuum and people with HIV (PWHIV) who are either in treatment gap, virologic failure, or lost to care. The study was designed to improve service organizations’ adoption of the use of SIMC to identify these cases, and to strategize ways to reengage them in care and improve treatment outcomes. The evaluation design assessed both the training and technical support process with service providers/staff and the health outcomes of PWHIV identified as out of care or not in virologic suppression. The study is particularly significant given the value globally of using nationally and locally available data monitoring systems to identify gaps in the HIV care continuum system to achieve significant improvements in population level health outcomes.
The study used a “hybrid type 3 mixed-method implementation study” design. However, this is not well defined when introduced, and the reader is left to infer what that means.
**Author’s answer:** As recommended, the hybrid type 3 mixed-method implementation study concept was incorporated when introduced in the section Study design. Changes are available bellow:

*This is a hybrid type 3 mixed-method implementation study that tests an intervention to integrate the CCM within public services for PLHIV, gathering information on the intervention and related outcomes. The intervention presents minimal risk associated with the strategies used. It was strongly encouraged by the STI/AIDS Program and adopted from a pilot intervention in a similar context setting [8]. The focus includes understanding the adoption of CCM, clinical impact, and changes in work processes.*

The study used well-accepted theoretical (REAIM) and methodological (PRISM) approaches to implementation research and evaluation in real world settings. The intervention process was well described and well-staged to improve implementation success, with repeated training and follow-up and multiple time point evaluation and outcome measures. Unfortunately, Figure 2 needs revision, because it contains too much text about the implementation design in font sizes and colors that (even in the TIFF file) are not sufficiently legible. This level of detail should be laid out in the text of the manuscript, leaving only the design outlines for the figure. Likewise, Figure 5 on the REAIM component findings is difficult to read, and would be better presented in the text of the manuscript as numbered or bulleted points below each of the REAIM constructs.
**Author’s answer:** - As recommended, Figure 2 was revised and the text about strategies applied was reallocated in the text of the manuscript. Changes are available bellow:

*Each of the three implementation phases included an individual technical visit to each service for approximately 4 h, followed by a 4-h face-to-face meeting within 60 days involving multiple services from the region (up to nine health services). ERIC strategies [13] applied in phases 1, 2, and 3 involved:*

*- Conduct educational outreach visits to train staff to answer questions related to the use of SIMC and train the team in the development of goals related to the CCM.*

*- Make training dynamic involving staff in SIMC use.*

*- Tailor strategies to address barriers and leverage facilitators in care flow.*

*- Gain and share local knowledge on CCM.*

*- Organize implementation team meetings to support and provide them protected time to reflect about CCM and share lessons learned.*

*- Promote adaptability, identifying ways CCM can be tailored to meet local needs.*

*- Facilitate problem solving related to CCM.*

*- Purposely reexamine the implementation to monitor progress and adjust clinical practices.*

Figure 5 was changed to Table 6, where we review and present the results for each REAIM constructs, improving the reading and understanding about how we achieve these results. Changes are available bellow:

*The results of the intervention are presented according to the dimension of the RE-AIM implementation science framework, as shown in Table 6.*

*Table 6. Intervention results according to the RE-AIM dimension*

| ***RE-AIM*** | ***Indicator*** | ***Level*** | ***N (%) or Results*** |
| --- | --- | --- | --- |
| ***REACH*** | *Patients with treatment gap reached through this intervention* | *Patients* | *94 (38.7%)^a^ patients from 243 identified.* |
|  | *Patients with virologic failure reached through this intervention* | *Patients* | *928 (78.1%)^b^ patients from 1,188 identified.* |
|  | *Patients with loss to follow-up reached through this intervention* | *Patients* | *1,552 (78.6%)^c^ patients from 1,975 identified* |
| ***EFFECTIVENESS*** | *Patient with treatment gap who were introduced to the treatment* | *Patients* | *47 (19.3%)^d^ started treatment from 243 reached.* |
|  | *Patient with virologic failure who had suppressed viral load* | *Patients* | *456 ^e^ (49.1%) patients had undetectable viral load from 928 patients reached.* |
|  | *Patient with loss of follow-up who restarted treatment* | *Patients* | *700^f^ (45.1%) patients restarted treatment from 1,552 reached* |
|  | *Strategies adopted in routine of services to monitor and reengage patients in care* | *Healthcare services* | *Improvements in healthcare services flows, described in Figures 6 and 7* |
| ***ADOPTION*** | *Of the 33 clinics invited, how many successfully implemented the clinical monitoring* | *Healthcare professionals* | *30 (90.9%) accepted from 33 invited* |
|  | *Perception of professionals about the integration of the clinical monitoring to the existing work* | *Healthcare professionals* | *69 (53.5) agreed or strongly agreed that they could easily integrate CCM before the intervention and 37 (75.5%) afterward* |
| ***IMPLEMENTATION*** | *Percent of services that developed the plan with goals related to CCM until the end of 2020* | *Healthcare professionals* | *27 (90.9%) healthcare services* |
|  | *Percentage of staff that believed that participating in the CCM is a legitimate part of their role* | *Healthcare professionals* | *32 (24.8%) agreed or strongly agreed to participate in CCM as part of their role before the intervention and 43 (87.8%) afterward* |
| ***MAINTENANCE*** | *Perception of professionals about continued support for the CCM* | *Healthcare professionals* | *115 (89.1%) agreed or strongly agreed that they will continue to support CCM before the intervention and 43 (87.8%) afterward* |
|  | *Staff believed that the CCM is worthwhile* | *Healthcare professionals* | *83 (64.3%) agreed or strongly agreed that CCM is worthwhile before the intervention and 34 (69.4%) afterward* |
|  | *Staff believed that they may modify how they work with the CCM* | *Healthcare professionals* | *94 (72.9%) agreed or strongly agreed that they may modify their work before the intervention and 39 (79.6%) afterward* |

*^a^Number of patients reached (47 started treatment + 47 refused treatment)/number of patients with treatment gap × 100.*

*^b^ Number of patients reached/number of patients with virologic failure × 100.*

*^c^ Number of patients reached (700 restarted and remained treatment + 138 restarted and stopped + 27 just reengaged follow-up+ 39 refused + 139 remained on LFU after several strategies + 509 outdated telephone numbers)/number of patients in LFU × 100.*

*^d^ Number of patients started treatment until the end of intervention/patients in treatment gap × 100.*

*^e^ Number of patients with undetectable viral load until the end of intervention/patients with virologic failure reached by intervention × 100.*

*^f^ Number of patients who restarted treatment until the end of the intervention/patients with loss of follow-up reached by the intervention × 100.*

The multilevel evaluation design is strong, including using the national electronic monitoring system (SIMC) to measure improvements in patient outcomes, the repeated listening sessions to document provider perspectives on using the system, documentation of strategies and service plans for reaching patients needing follow-up, and plans for sustaining use of the monitoring system. Additionally, a pre/post survey (NoMAD) was used with providers at the 30 participating service organizations to measure their perspectives on adopting routine activities to incorporate and sustain use of the clinical monitoring system as part of their routine HIV care continuum activities. However, a better description of this questionnaire would be beneficial up front, or at least reference to Table 7 for full content of the survey.

**Author’s answer**: A better description of the questionnaire was incorporate in Materials and Methods section, adding more two references related to the questionnaire. The paper which presents the cross-cultural adaptation of the NoMAD questionnaire to Brazilian Portuguese was published in October and the reference number 15 was updated. Changes are available bellow:

*During the pre-implementation, service professionals were invited to participate and answer the Normalization Measure Development (NoMAD) questionnaire to identify contextual factors impacting the implementation of the CCM. This questionnaire is based on the normalization process theory (NPT) and determines the collective behavior for the incorporation of complex interventions in practice. According to the NPT, the implementation of new practices in healthcare services is dynamic and dependent on the coordinated and collective behavior of individuals who work within the limits of healthcare contexts [11]. NoMAD has 23 items distributed in four constructs: coherence, participation or cognitive engagement, collective action, and reflective monitoring [12].*

The methods for calibrating treatment gaps, virologic failure and lost to care using the SIMC appear reasonable and rigorous, including steps to remove duplicates and ineligible patients from the list of target patients. However, the presentation in Table 2 is difficult to follow in terms of conditions of the patients in the database before the intervention and/or before/after sorting for targeted services.
**Author’s answer:** As recommended, Table 2 was improved to better understanding. We transform Tables 2, 3 and 5 in just on table (Table 2) because all of these Tables comprised ineligibles patient from different reports, but with similar situations (p.ex.: Death or duplicate records). Changes are available bellow:

***Table 2. Patients’ status in SIMC report after analyses by the healthcare services***

| ***Status*** | ***Gap (%)*** | ***Failure (%)*** | ***LTFU (%)*** |
| --- | --- | --- | --- |
| *Death* | *98 (7.9)* | *29 (1.8)* | *1126 (60.8)* |
| *Duplicate records* | *955 (77.4)** | *60 (3.8)#* | *236 (12.7)* |
| *Transferred* | *13 (1.1)* | *98 (6.2)* | *253 (13.7)* |
| *No medical record in the healthcare service* | *24 (1.9)* | *44 (2.6)* | *198 (10.7)* |
| *HIV negative* | *144 (11.7)* | *1 (0.1)* | *38 (2.1)* |
| *VL suppression before review* | *NA* | *150 (9.6)* | *NA* |
| ***Total*** | *1430 (100)* | *1570 (100)* | *3819 (100)* |
| ***Excluded*** | ***1,234 (81.2)*** | ***382 (24.3)*** | ***1,851 (48.3)*** |
| ***Included/ eligible*** | ***243 (18.8)*** | ***1,188 (75.7)*** | ***1,968 (51.7)*** |

**Patients had started ART but appeared in the report due to duplicate records not identified by the interaction of databases for laboratory examinations and drug dispensation.*

*#Patients had viral load suppression but had duplicate records in the laboratory system.*

*^Patients had restarted treatment but had duplicate records in the HAART system.*

Findings related to patient/client health appear to be robust and reliable, given that they are derived from the ongoing national monitoring system from which patients needing extra services were identified. Also, strategies both to indicate and to engage PLHIV who are in treatment gap, virologic failure, or lost to care are well described. However, there are two Tables 6 presenting these data, which should be differently numbered.
**Author’s answer:** As recommended, The Table/Figure number was corrected.

While the study was strong in relation to replication of a rigorous intervention implementation and evaluation design systematically conducted in 30 different service settings, it might have been strengthened and the conclusions more strongly supported by comparing patient outcomes in similar comparison communities and/or service organizations that did not receive the intervention training and support program, in addition to those that did. This limitation to the study design should at least be mentioned in the Limitations section.
**Author’s answer:** The limitation section was updated to include to approach this limitation in design. Changes are available bellow:

*The study was not designed to compare the results with a similar control group. Unfortunately, it is impossible to return to the SIMC to compare the results achieved in the regions with intervention with other regions or the whole state at the same time period. A control group, although not feasible regarding the changes in the workflow and process at the service level, could be included with respect to the SIMC outcomes related to treatment gap, virologic failure, and loss of follow-up. In contrast, this intervention, supported by the STI/AIDS Program from the State of Sao Paulo achieved important results in patients, healthcare services, and program level, when we compared the pre-and post-intervention outcomes. Additionally, we hope that these results will support and improve the reports available in the system.*

The pre/post analyses of responses on the NoMAD survey need further analysis. While the comparison statistics seem appropriate, the very high attrition rate (65%) raises the question of attrition bias in the follow-up sample and challenges confidence in the strength of the findings. This should be addressed by explaining better who did not respond to the follow-up and possible reasons they did not. The current comment, that the non-responders are likely to be service staff not responsible for these tasks, is insufficient. Additionally, this low response rate to the follow-up NoMAD survey should be mentioned in the Limitations section.
**Author’s answer:** The limitation section was updated to include to approach this limitation about low response rate to NoMAD questionnaire. Changes are available bellow:

*The number of providers that answered the NoMAD questionnaire at the end of the intervention decreased compared with the number at the start of the intervention. This could be related to two factors observed but not measured during the intervention: 1. At the beginning of the intervention, the managers usually asked for all healthcare service providers to answer the questionnaire, even before defining whether all professionals would participate in the intervention. 2. The CCM activities were centered on specific members of each healthcare service and the majority of respondents of the questionnaire at the end of the intervention.*

The protocol has been made available through the Brazilian clinical research registry. However, it is not clear if all data are available to the public.
**Author’s answer:** The data are available to the public. The status of section data availability was change to “all data are fully available…” and was add the following section on methods:

*“Data were analyzed using the Software Stata/IC 14.0^®^ and are available at http://www.saude.sp.gov.br/centro-de-referencia-e-treinamento-dstaids-sp/publicacoes/publicacoes-download”.*

The whole manuscript, including Figures, would benefit from additional proofreading for grammatical and usage issues or missing words. Particular challenges with clarity include the last sentence before Table 6, and sections of the Discussion, especially the 4th, 5th, and 6th paragraphs, among other places here and there throughout the manuscript.

**Author’s answer:** To attend this solicitation and editor’s recommendation, the manuscript was reviewed by EDITAGE.

**Reviewer #2**: Thank you for the opportunity to review this manuscript. The authors present an evaluation of a technical support strategy to increase uptake of clinical monitoring of patients with HIV along the treatment cascade in Sao Paulo, Brazil. They used a mixed methods approach, combining pre/post questionnaires with providers, reviews of clinical databases to track patients through the care cascade, and notation of strategies adopted by providers to support uptake of CCM. Questionnaire results suggest that providers are enthusiastic about CCM and plan to sustain it. However, the patient-level analysis is not informative as to the effects of the implementation strategy, as there are no comparators (either pre-implementation or control) against which to judge relative uptake of CCM and its subsequent effects. The paper contains many useful pieces of information but is generally dense and the findings are difficult to interpret. I would recommend the authors consider a quasi-experimental evaluation approach to judge the effects of the technical support implementation strategy on CCM uptake and patient outcomes (e.g., tracking rates of virologic failure among implementing facilities pre- and post-implementation), or reframe this paper as a case study. See below for some major critiques:
- Perhaps most importantly, it is not clear what the data in Tables 2-6 tell us about the effects of the technical support implementation strategy on CCM usage or patient-level cascade outcomes. Table 6 comes closest by comparing patients pre- and post-implementation, but here the focus is on comparing patient characteristics, not patient probability of restarting therapy conditional on being in the pre- or post-implementation period. Useful patient-level estimates of effectiveness would answer questions like, “What proportion of clinic patients are in virologic failure pre-implementation, vs. what proportion are in virologic failure post-implementation?” Ideally, comparisons like this would also include a pre-post comparison with one or more control facilities.

**Author’s answer:** The tables 2,3 and 5 demonstrated the results from the steps to remove duplicates and ineligible patients from the list of target patients, essentials to understand calculate the reach from REAIM framework. As cited in a previous answer to the other reviewer, these three tables were transformed in just one (Table 2) to improve comprehension.

Table 4 and 6 demonstrated the profile of the patients assessed by the health care services and helps to understand possible individual factors that could interfere in the results achieved, as discussed in the Discussion section. E.g.: at least 50% of patients with virologic failure did not have adequate % of pills dispensed in the last year, signalizing low adherence.

**Author’s answer:** Tables 4 and 6 (renamed to Tables 3 and 4) tell us to much about the individual factors that could affect the intervention results, so we decide to maintain these table just with key patients’ characteristics that could explain the results founded. Additionally, the limitation section was updated to include the limitation related to the lack of a control group.

- It is unclear how the PRISM framework was incorporated.

**Author’s answer:** The use of PRIS was clarified in the method section. Changes are available bellow:

*Based on the Pragmatic, Robust Implementation and Sustainability Model (PRISM) for translating research into practice [9], this conceptual framework was applied to identify which recipients could influence the CCM reach, adoption, implementation, maintenance, and effectiveness. Figure 1 shows contextual factors and evaluation of intervention adoption and effectiveness according to the dimensions of the RE-AIM planning and evaluation framework [10].*

- Several of the RE-AIM components are inappropriately mapped to study indicators. For example, Reach implies an estimate of the number of patients served divided by the number of eligible patients. In this study, your reach indicators appear to be the number of patients still remaining in each cascade bucket (e.g., virologic failure) after data cleaning.

**Author’s answer:** The RE-AIM indicators were recalculated considering the reviewer comment and the literature. Also, Figure 1 is now presenting the formula used to measure the dimension Reach from RE-AIM for treatment gap, virological failure and LFU and Table 6 synthetize all the results considering the dimensions of this framework.

- Figure 2 makes it appear that the NoMAD questionnaire was only given at pre-implementation, but Methods Section 3 implies that NoMAD was given pre- and post-implementation to track changes in provider perspectives. Please clarify in Figure 2.

**Author’s answer:** As recommended, the response to NoMAD was added in the post-implementation step in Figure 2.

- Regarding the pre/post CCM implementation questionnaire analysis – do you treat the pre- and post- observations as independent, or do you account for observations of the same provider at pre- and post (paired analysis)? This is not clear.

**Author’s answer:** The analysis considered the sample as independent because this is an implementation science study and assessed real life settings. This information was added in the section 3. Professionals’ perspectives of the implementation. Changes are available bellow:

*To evaluate significant differences from a statistical point of view on professional’s perception regarding the CCM implementation, responses to the items of the questionnaire, for pre- and post-implementation, were compared as independent samples using the Mann–Whitney U test.*

- Methods/Population section – were facilities randomly sampled on the basis of patient volume lost to care, or purposively selected?

**Author’s answer:** The facilities were selected based on patient’s volume in the SIMC reports. This section was clarified in Population section. Changes are available bellow:

*The services were selected based on the volume of patients lost from the continuum of care in April 2019, consisting of 1,186 patients with treatment gap (11.5% of the State), 1,328 patients with virologic failure (14.8% of the State), and 3,449 patients who were lost to follow-up (12.5% of the State) [3].*
