# Supplementary material for: Improving the continuum of care monitoring in Brazilian HIV healthcare services: An implementation science approach
Source: PLoS One. 2021 May 10;16(5):e0250060. doi: 10.1371/journal.pone.0250060 (PMC8109816; doi:10.1371/journal.pone.0250060)
Supplement: Attachment — Submitted filename: Response to Reviewers.docx [file pone.0250060.s002.docx]

Dear Editor of PLOS ONE,

Thank you for the comments and suggestions made to the manuscript “Improving the Continuum of Care Monitoring in Brazilian HIV Health Care Services: An implementation science approach”, submitted to PLOS ONE. The suggestions and comments contributed to improve our paper with a view to publication in this Journal. Below we present our responses to the comments from editors and reviewers, as well as the changes made, identified in the revised manuscript.

Best regards

Ana Paula Loch

**Editors’ comments:**

Reviewer #1: Most of the responses to the previous critiques are thorough and adequate to improve and address problems of the previous manuscript. The paper has been greatly strengthened and clarified, reads much better, provides important information regarding the intervention, and makes an important contribution to implementation science.

A few points remain to be clarified or improved before publication of the paper. A reviewer requested an explanation of “hybrid type 3 mixed method implementation study.” That term is still not defined, though more detail is provided on the study design. A simple statement that specifically defines “hybrid type 3” would be extremely valuable.

**Author’s answer:** Thank you. The statement that defines the concept of hybrid type 3 was provided as suggested.

Regarding the concern that there were significantly fewer time-2 survey responses on the NoMAD and implications of this on reported findings in the “Professionals’ Level” section: If it is the case that professionals took the baseline survey who were not intended to participate in the intervention, and only those who received the intervention responded to the follow-up survey, why weren’t the baseline surveys of the unintended for intervention dropped from analysis, and only the baseline and follow-up surveys of those professionals relevant to or receiving the intervention used in these analyses? It seems likely to have affected all of the analyses of statistical significance if people who we not directly related to or involved in the intervention were included in analyses reported in both the text and Tables 5 and 6. The explanation provided in the Discussion section for the reduced follow-up response rate does not change the problem with how the data are reported in the results section.

**Author’s answer:** All the professionals those took survey in baseline had the intention to participate. This study was on real-life and it was conditioning to the local reality, also affected by the pandemic. Additionally, to avoid embarrassment of participants, the survey was done anonymously, which limits the paired comparison. A better explanation was done about this limitation on referred section and on item “Professionals’ perspectives of the implementation”.

There still are many grammatical issues with the language. It may need additional editing by a native English speaker. (E.g., see line 314: “…among 234 patients had treatment gap…” among several others throughout the paper.)

**Author’s answer:** The manuscript was reviewed by EDITAGE after we consider all comments from this review.

Other smaller points:

1. All figures are still fuzzy. Will they be clear in print?

**Author’s answer:** yes, we printed a version to test and it is clear.

2. It is not clear what the caption to Fig. 1 refers to since it does not seem to correspond to any lettering in the figure and there is no indication of a footnote in the image.

**Author’s answer:** The caption referred to the formulas used to calculate the indicator “Proportion of patients in target population reached through this intervention”. Considering this comment, we made available the formulas in the text, removing the caption from the figure 1. Thank you.

3. Itemized list of ERIC strategies starting line 166-175 – change verbs to “ing” form to follow the phrase “…1, 2 and 3 involved:” e.g., - conducting…; - making… etc. Or else, complete the phrase as a sentence: “…1, 2, and 3 involved the following tasks.”

**Author’s answer:** The text was changed as suggested. Thank you

4. In Figure 3, the lightening bolts are different colors. However, if printed in black and white, this cannot be distinguished. I recommend using different symbols for the different problems (treatment gap, virologic failure, loss to follow-up).

**Author’s answer:** The figure was changed, using different symbols as suggested.

5. The ^ item in Table 2 referenced in the footnote is not evident in the table.

**Author’s answer:** The item was added in the table as suggested.

6. Check/correct periods and commas in Table 3 title and row “>80%”. Check elsewhere in the manuscript as well, e.g., line 346.

**Author’s answer:** The periods and commas were reviewed in all manuscript as suggested.

7. Delete blank column in Figure 7.

**Author’s answer:** The blank column was removed as suggested.

**Reviewer #2:** Thank you for the opportunity to re-review this manuscript. The authors have strengthened the manuscript since the first review round, and the paper is now much clearer. Methodologically, I think the study is still very limited in terms of its ability to assess the effectiveness of the strategies to increase CCM adoption among the health facilities in the sample, given that they had no control group. The authors acknowledge this and other limitations at the end of their discussion section. Nonetheless, the paper presents interesting data that will be useful for colleagues implementing similar interventions.

- Page 12, lines 272-274: Please clarify the denominators used in the proportions here. For example, were 11.5% of the State population of Sao Paulo in treatment gap? Or 11.5% of PLHIV in Sao Paulo who were in the CCM system?

**Author’s answer:** The denominators were clarified in this section as suggested.

Also, should these population totals (e.g., 1186 patients with treatment gap) align with those from the Results section (Page 14 line 300, 1430 patients in treatment gap)? If not, please explain the discrepancy.

**Author’s answer:** No, this totals are not align because 1186 refers to the beginning of the intervention, but as explained in Figure 2, in the phase 2 we identified new cases in the report, so this number increased. A new sentence was added in this section to clarify this difference.
